# Tendinous Signal Alterations on MRI in the Asymptomatic Elbow: A Retrospective Cross-Sectional Study

**DOI:** 10.5334/jbsr.3651

**Published:** 2024-07-13

**Authors:** Bjorn Valgaeren, Elyn Van Snick, Bart Claikens

**Affiliations:** 1 Department of Radiology, KU Leuven, Herestraat 49, Leuven 3000, Belgium; 2 Department of Radiology, Universitair Ziekenhuis Brussel, Brussels Health Campus, Laarbeeklaan 101, Jette 1090, Belgium; 3 Department of Radiology, General Hospital Ostend, Gouwelozestraat 100, Ostend 8400, Belgium

**Keywords:** Magnetic resonance imaging, asymptomatic, elbow, prevalence, tendinosis, tendinopathy

## Abstract

**Objectives:**

It is clinically relevant to prevent overtreatment of tendinopathy diagnosed solely on imaging. Therefore, the prevalence of presumable asymptomatic signal changes in the common flexor origin, biceps insertion, brachialis insertion, and triceps insertion were assessed.

**Materials and methods:**

Two hundred and five magnetic resonance imaging (MRI) exams of the elbow with coronal and axial fat-saturated fluid-sensitive sequences between January 1, 2018 and July 31, 2022 were retrospectively identified in our center.

Two radiology residents reviewed the exams independently. The elbow tendons were given a score from 0 to 4. Score 0: no signal abnormality; score 1: increased T2-weighted signal around the tendon; score 2: increased T2-weighted signal compared to muscle within the tendon; score 3: partial tear; and score 4: complete tear.

**Results:**

The common flexor tendon showed signal alterations in 8% of patients; nine patients had an increased signal around the tendon, and eight patients had an increased signal within the tendon. Three patients (1.5%) had an altered signal intensity in the biceps tendon. All triceps tendons showed a linear hyperintense signal, suggesting that it is physiological. There were no partial or complete tears. No signal abnormalities were noted in the brachialis tendon among all patients.

**Conclusion:**

The prevalence of presumable asymptomatic signal alterations seen in the common flexor origin on MRI is not negligible; therefore, clinical correlation is advised to prevent overtreatment of tendinopathy in these cases. No partial or complete tears were seen.

## Introduction

Tendinopathy of the elbow, of which lateral tendon pathology is most common, can have several causes but is most frequently the result of overuse, with repetitive microtrauma leading to microtears and subsequent fibrosis and angiofibroblastic tendinosis. Eventually, macroscopic tears might also occur [1, 2]. Elbow tendinosis affects a wide range of patients, with both middle-aged people, athletes, and certain professions at risk, resulting in significant morbidity. Repetitive motions, smoking, diabetes, and obesity contribute to a poor prognosis [2].

Magnetic resonance imaging (MRI) is considered the gold standard for the evaluation of elbow joint pathology and elbow tendinosis [3]. Histopathological changes in tendinosis are reflected by an increase in MRI signal intensity both on T1- and T2-weighted images [1, 4]. Other findings in tendinopathy are a thickened and irregular aspect of the enthesis, peri-entheseal soft tissue oedema, and adjacent bone marrow oedema of bony erosions [5].

Different studies showed asymptomatic signal changes in the origin of the extensor carpi radialis brevis (ECRB) and common extensor tendon with varying prevalence (11%–55%) [4, 6–8]. These findings became more prevalent with older age [4].

Tendinopathy of the distal biceps brachii tendon is rarer [9]. Asymptomatic signal intensity changes range from 0.6% to 1% in the literature [10, 11]. The prevalence of asymptomatic changes in the common flexor origin reached up to 3% [10].

A prospective study in asymptomatic volunteers demonstrates intratendinous signals similar to muscle in up to 96% of the participants in the distal triceps and 94% in the brachialis tendon. The authors concluded that these higher signals would be normal because they were present in the same amount in all age categories. Only 1% of the triceps and 2% of the brachialis insertions showed intratendinous signal abnormalities [11].

### Objective

**Figure 1:**
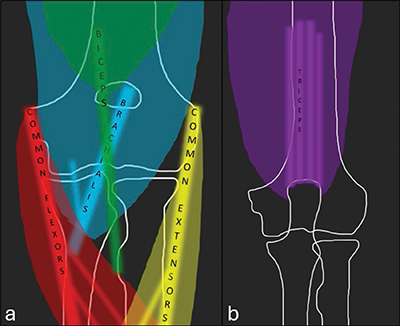
Anterior **(a)** and posterior **(b)** coronal projections of the major tendons and muscle bellies around the elbow.

The objective of this study was to assess the prevalence of presumable asymptomatic signal changes in the common flexor origin, biceps insertion, brachialis insertion, and triceps insertion (Figure 1).

## Material and Methods

The approval of the ethics committee at our institution was obtained. Informed consent was waived due to the retrospective nature of the study.

### Patient selection

All patients who underwent an MRI exam of the elbow between January 1, 2018 and July 31, 2022, were retrospectively identified in our center.

Patients were excluded if at least one of the common flexor, distal biceps, brachialis, and triceps tendons was incompletely depicted. Exclusion was also done if patients exhibited symptoms of pathology related to these tendons, including ulnar-sided elbow pain, pain over the olecranon, painful stress tests, painful palpation over the tendons, isolated muscle atrophy, muscle weakness, visible tissue swelling, and skin redness. This was determined based on the clinical information provided on the request and by reviewing the patient’s electronic medical record. If the request was incomplete or not readable, the examination was excluded.

Patients with a prior history of systemic inflammatory disease, trauma, tear, or surgery to the tendons under investigation were also excluded. Lastly, if patients underwent multiple MRI exams during the set period, only the most recent exam was included (Table 1).

**Table 1:** Inclusion and exclusion criteria with the number of excluded examinations (*n*).

INCLUSION	EXCLUSION (*N = 172*)
Between January 1, 2018 and July 31, 2022	Symptoms possibly related to the investigated tendons (*n = 106*)
Elbow MRI with fat-saturated axial and coronal fluid-sensitive sequences	Absence or insufficient clinical information (*n = 27*)
	Minors (*n = 12*)
	Double examinations (*n = 12*)
	Insufficient image quality (*n = 11*)
	Previous surgery (*n = 3*)
	Acute trauma (*n = 1*)

### Technical details

All exams were performed on a Siemens Aera 1.5 Tesla system. The images had to include axial and coronal T2-weighted turbo spin echo sequences with fat saturation, of which the most had the following parameters: repetition time (TR) of 3500 ms, time to echo of (TE) 38 ms in the coronal plane and 78 ms in the axial plane, and coronal slice thickness of 2.5 mm compared to 4 mm in the axial plane.

The field of view (FOV) was set to 128.1 mm × 100 mm with a pixel size of 0.33 mm × 0.26 mm for both coronal and axial images.

In a few cases, when T1-weighted images before and/or after contrast were available in the picture archiving and communication system (PACS) for a particular examination, they were not reviewed by the investigators.

All examinations were archived on a PACS (Synapse; Fujifilm, Lexington, USA) and reviewed on a six-megapixel medical display (Barco, Kortrijk, Belgium).

### Scoring system

The exams were independently reviewed by two radiology residents. The elbow tendons were given a score from 0 to 4, similar to a method established by De Grove et al. [12]: score 0: no signal abnormality; score 1: increased T2-weighted signal around the tendon; score 2: increased T2-weighted signal compared to muscle within the tendon; score 3: partial tear; and score 4: complete tear [11].

A consensus between the two readers was reached if they scored differently under the supervision of a senior radiologist experienced in musculoskeletal radiology. Cohen’s kappa coefficient was calculated to evaluate the interobserver reliability.

## Results

Eventually, 205 patients were included in the study (81 male, 124 female), with a mean age of 50 (18–84). 33 (16%) adults are younger than 40, 136 (66%) are included in the category of 40–59, and 36 (18%) are older than 60.

Twenty patients showed abnormal signal intensities (score 1 or higher) in one of the tendons (Table 2). One in the age category of 18–39, fifteen between 40 and 59, and four older than 60 (proportionally 3%, 11%, and 11%).

**Table 2:** Distribution of findings across all 205 elbows.

	SCORE 0	SCORE 1	SCORE 2	SCORE 3	SCORE 4
**Biceps**	202 (98.5%)	1 (0.5%)	2 (1%)	0	0
**Brachialis**	205 (100%)	0	0	0	0
**Common flexor**	188 (92%)	9 (4%)	8 (4%)	0	0
**Triceps**	0	0	205 (100%)	0	0

No individual had more than one abnormality detected. There were no partial or complete tears. No signal abnormalities were noted in the brachialis tendon.

**Figure 2:**
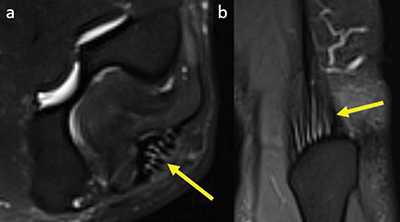
Axial **(a)** and coronal **(b)** fat-saturated T2-weighted image at the level of the triceps tendon insertion with a physiological linear T2-hyperintense signal compared to muscle parallel to tendon fibers, score 2.

**Figure 3:**
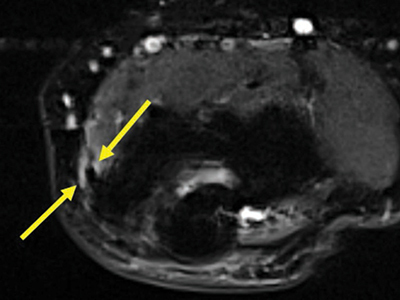
Axial fat-saturated T2-weighted image through the medial flexor origin with a peritendinous T2-hyperintense signal compared to muscle, score 1.

**Figure 4:**
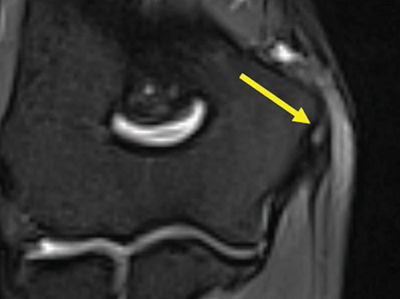
Coronal fat-saturated T2-weighted image through the medial flexor origin with an intratendinous T2-hyperintense signal, score 2.

Multiple linear T2-hyperintensities were seen distally in the triceps tendon parallel to its fibers among all patients (Figure 2). The common flexor tendon showed signal alterations in 8% of patients; 4% of them had an increased signal around the tendon (score 1) (Figures 3) and 4 % of the patients had an increased signal within the tendon (score 2) (Figure 4).

**Figure 5:**
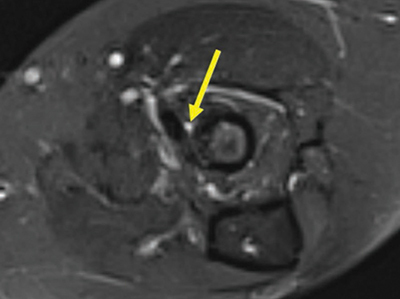
Axial fat-saturated T2-weighted image at the level of the biceps tendon insertion with a T2-hyperintense signal next to it, score 1.

**Figure 6:**
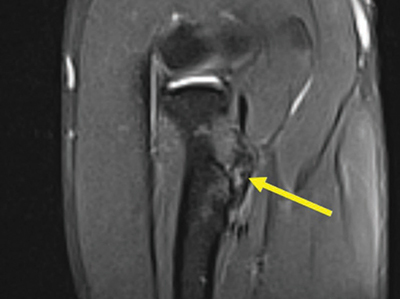
Coronal fat-saturated T2-weighted image at the level of the biceps tendon insertion with an irregular T2-hyperintense signal within, score 2.

Three patients (1.5%) had an altered signal intensity in the biceps tendon. One patient had a score of 1 (Figure 5), and the other two had a score of 2 (Figure 6).

The interobserver reliability of all tendons combined between the two investigators was excellent (Cohen’s κ = 0.97; CI 95% [0.95; 0.99]). There were no differences between the observers in giving a score of 3 or 4 on any of the tendons.

In one examination, the flexor origin was scored 2 by the first reader and 0 by the second, and 2 and 1 subsequently in a different examination. A score of 0 and 1 was sometimes incongruent in scoring the flexor origin and biceps insertion.

## Discussion

MRI is an important imaging tool for evaluating elbow pain and elbow tendinosis. However, several studies have exhibited that abnormalities visualized on MRI do not always correlate with patients’ symptoms [4, 10–13].

This study showed that 10% of patients had abnormal signal intensity in at least one of the elbow tendons under investigation. To the best of our knowledge, none of these patients experienced related symptoms. This is an important finding because misinterpretation of these presumable asymptomatic tendon abnormalities visualized on an MRI may lead to overdiagnosis and overtreatment.

All observed signal abnormalities were given a score of 1 or 2. Therefore, we assume that asymptomatic tears would be rare in the observed tendons, and they would normally be accompanied by symptoms.

The common flexor tendon was the tendon in which alterations of signal were most frequently observed (8%). Half of these abnormal signals occurred in the tendon itself, mimicking a tendinopathy, similar to the findings in the literature (3%) [11].

Only a small percentage of patients (1.5%) had abnormal signal intensities in the biceps tendon. This prevalence is in line with earlier reports [10, 11].

Pathology of the triceps [10] is rare, and it is extremely rare in the brachialis tendon [14]. Sevag et al. suggested that small linear signals are part of the normal tendinous structure of the triceps insertion [11]. These were present in our study too and are considered normal. No other signal abnormalities were noted in the triceps or brachialis insertion across all patients in this study, suggesting the pathology of these tendons to be extremely rare in presumable asymptomatic individuals too.

Pathological signal changes occurred most of the time in middle-aged adults and seniors, with a proportion of 11% each, compared to 3% in the junior group in our study, which may support the theory of a higher tendon signal in presumable asymptomatic age-related degeneration [4, 11].

### Limitations

The main limitation of this study is its retrospective character. One of the exclusion criteria—absence of symptoms—was based on the clinical information provided on the request and the available information in the patient’s electronic medical record. There is a possibility that some patients participating in the study may have had a history of symptoms or trauma that had not been documented. In patients who received treatment for their tendinopathy, signal alterations on the MRI might still persist even after symptoms have subsided [13].

Furthermore, these people underwent an examination because they had symptoms of other diseases, which might have been a risk factor for developing disease in the tendons we evaluated, possibly causing susceptibility bias.

An association between lateral pathology and the investigated tendons could not be ruled out either.

Symptoms of other diseases might have made patients less aware of the ongoing symptoms of concomitant tendon pathology.

Additionally, possible treatment for any other existing tendinopathy was not taken into account. This might have potentially obscured symptoms that could have been caused by tendinopathy of the investigated tendons.

Lastly, no other risk factors for acquiring a tendinopathy, like sports or jobs requiring repetitive movement of the investigated tendons, were considered.

## Conclusions

Despite some limitations, we can conclude that: the prevalence of presumable asymptomatic signal alterations seen in the common flexor origin on MRI is not negligible; therefore, (1) clinical correlation is advised to prevent overtreatment of tendinopathy in these cases; (2) presumable asymptomatic signal alterations in the distal m. biceps brachii tendon can rarely be seen, in line with the existing literature; (3) asymptomatic tears of the common flexor origin, biceps insertion, brachialis insertion, and triceps insertion were not detected, therefore tears might prompt treatment; and (4) presumable asymptomatic tendinopathy of the brachialis and triceps brachii insertion are expected to be extremely rare.

## Data Availability

The data that support the findings of this study are available from the corresponding author, BV, upon reasonable request.
